# Report from the First Snake Genomics and Integrative Biology Meeting

**DOI:** 10.4056/sigs.3106480

**Published:** 2012-09-24

**Authors:** Todd A. Castoe, Edward L. Braun, Anne M. Bronikowski, Christian L. Cox, Alison R. Davis Rabosky, A.P. Jason de Koning, Jason Dobry, Matthew K. Fujita, Matt W Giorgianni, Adam Hargreaves, Christiaan V. Henkel, Stephen P. Mackessy, Denis O’Meally, Darin R. Rokyta, Stephen M. Secor, Jeffrey W. Streicher, Kenneth P. Wray, Ken D. Yokoyama, David D. Pollock

**Affiliations:** 1 Department of Biochemistry & Molecular Genetics, University of Colorado School of Medicine, Aurora, CO, USA; 2 Department of Biology, University of Florida, Gainesville, FL, USA; 3 Department of Ecology, Evolution, and Organismal Biology, Iowa State University, Ames, IA, USA; 4 Department of Biology, University of Texas at Arlington, Arlington, TX, USA; 5 Museum of Vertebrate Zoology, University of California, Berkeley, CA, USA; 6 Amplicon Express, Pullman, WA, USA; 7 Department of Organismic and Evolutionary Biology, Harvard University, Cambridge, MA, USA; 8 Department of Genetics and Molecular Biology, University of Wisconsin Madison, Madison, WI, USA; 9 School of Biological Sciences, Bangor University, Bangor, UK; 10 LZF-screens BV and Institute of Biology, Leiden University, Leiden, NL; 11 School of Biological Sciences, University of Northern Colorado, Greeley, CO, USA; 12 Institute for Applied Ecology, University of Canberra, Canberra, AUS; 13 Department of Biological Science, Florida State University, Tallahassee, FL, USA; 14 Department of Biological Sciences, University of Alabama, Tuscaloosa, AL, USA

## Abstract

This report summarizes the proceedings of the 1st Snake Genomics and Integrative Biology Meeting held in Vail, CO USA, 5-8 October 2011. The meeting had over twenty registered participants, and was conducted as a single session of presentations. Goals of the meeting included coordination of genomic data collection and fostering collaborative interactions among researchers using snakes as model systems.

## A community using snakes as model systems

Snakes are gaining importance as model systems for a diversity of research. They are valued models for studying extreme physiological and morphological plasticity, evolutionary ecology, molecular evolution, developmental biology, and venom evolution. Despite the importance of snakes as models for basic and biomedical research, there is little known about the genomes of snakes, and there are minimal genomic resources currently available. These limitations, however, will soon be lifted as numerous groups are making progress in establishing complete snake genomes and genomic resources, thereby enabling numerous new areas of research utilizing snakes.

The first Snake Genomics and Integrative Biology meeting was held October 5-8, 2011 in Vail, Colorado USA to bring together an international collection of researchers from diverse backgrounds that share a common interest in snake genomics and comparative biology. A core aim of the meeting was to showcase ongoing and potential projects that utilize (or aim to utilize) snake genomic and transcriptomic data for integrative and comparative biology. The meeting was organized around the goals of catalyzing collaborative research, identifying shared interests in research and data collection, and coordinating among research groups to maximize scientific impact, collaboration, fundability, and utility of data generated to the community. Additional goals of the meeting included the coordination future goals and integration with the Genome10K project and other planned snake genome projects.

## Snake genomics

**David Pollock (University of Colorado School of Medicine, USA)** opened the meeting by detailing the many ways in which a broad understanding of snake genomics can contribute to a wide diversity of research efforts in applied and theoretical biology. **Todd Castoe (University of Colorado School of Medicine, USA)** updated the group on the progress of sequencing the genome of the Burmese Python (*Python molurus bivittatus*). As part of this project, Castoe showed data demonstrating how extensive changes in gene expression accompany the extreme physiological remodeling of the python when fed a large meal after being fasted. **Christiaan Henkel (ZF-screens BV and Leiden University, NL)** updated the group on the status of the King Cobra genome (*Ophiophagus hannah*), including analyses of the copy number of genes encoding toxic venom proteins. Henkel also discussed analyses he and colleagues have done on a lower-coverage draft sequence of the genome of the blindsnake (*Typhlops murius*). **Matt Giorgianni (University of Wisconsin – Madison, USA)** discussed plans to sequence the genome of the Western Diamondback Rattlesnake (*Crotalus atrox*) in order to study the evolution of venom-related genes. **Adam Hargreaves (Bangor University, UK**) discussed research on snake development and pigmentation, and the generation of draft genome sequences of Corn Snake (*Pantherophis guttatus*) and Saw-scaled Viper (*Echis coloratus*) as well as transcriptomic resources for these and other species. **Jason Dobry (Amplicon Express, USA)** discussed methods for utilizing the custom-made PMB-*Python molurus* bacterial artificial chromosome library for studying specific sets of genes, as well as assisting in the whole genome assembly. **Edward Braun (University of Florida, USA)** addressed the potential for including reptilian genomics in science pedagogy. In addition to examples of successes of the concept, Braun presented the group with the open question of whether genome annotation could be accomplished through pedagogical interactions with undergraduate classes.

There is little published information on the structure and function of snake genomes, although data shown by contributors at this meeting provided an exciting window into the apparently dynamic structure of snake genomes. **Matthew Fujita (Harvard University, USA)** discussed the evolution of genomic nucleotide content and isochore structure in vertebrates, and showed new data on isochore structure in snakes. **Todd Castoe (University of Colorado School of Medicine, USA)** showed data describing the evolution genomic repeat element landscapes in snakes, including evidence of a shift in transposable element activity within snakes. **Ken Yokoyama (University of Colorado School of Medicine, USA)** presented an overview of cis-regulatory element characteristics in vertebrates, and evidence for the relative uniqueness of features of snake cis-regulatory elements based on analyses of the python. **Denis O'Meally (University of Canberra, AUS)** discussed the evolution of sex chromosomes in reptiles, highlighting the insights into sex-specific regulation and dosage compensation that the independently evolved sex chromosomes of snakes can provide. **Jason de Koning (University of Colorado School of Medicine, USA)** discussed methods for analyzing protein-coding genes to detect positive selection indicative of adaptation, including pitfalls of existing approaches and suggestions for alternatives that are planned for analysis of the python genome.

## Evolution and genetic basis of important traits in snakes

Genomic approaches to the study of color and pigmentation patterns also figured prominently at the meeting. Color pattern is important because it is intimately linked to snake ecology, as pigmentation controls predator avoidance through crypsis, regulation of body temperature, and warning coloration in venomous coral snakes and their harmless mimics. **Adam Hargreaves (Bangor University, UK)** presented data on the genomics and transcriptomics of pigmentation pattern development in multiple snake and lizard species, with foci on Corn Snakes, Saw-scaled Vipers, and Leopard Geckos. Collaborators **Christian Cox (University of Texas at Arlington, USA)** and **Alison Davis Rabosky (University of California Berkeley, USA)** focused on the role of color in mimicry systems, including their work on the genetics of red and black pigmentation in the mimetic Ground Snake (*Sonora semiannulata*). They then presented an ongoing transcriptomics project to identify expression differences of color genes in multiple color morphs of mimetic species across the snake radiation. These comparative genomic and transcriptomic projects will be key to identifying the genes that underlie color pattern in snakes, furthering our understanding of how and why color pattern evolves over time and space. **Jeffrey Streicher (University of Texas at Arlington, USA)** discussed the difficulties in identifying evolutionary lineages and population differentiation in brightly colored coralsnakes from the United States and Mexico. He demonstrated the use of next-generation sequencing to identify microsatellite loci rapidly, and by combining these data with DNA sequences, venom variation, and morphological data, was able to better define evolutionary lineages in these venomous snakes.

**Anne Bronikowski (Iowa State University, USA)** presented data for the differential life history patterns among proximal populations of garter snakes, and how she was using these to study the tradeoffs between and consequences of these strategies at the molecular level using transcriptomic data. **Stephen Secor (University of Alabama, USA)** discussed the adaptive relationship between snake feeding habits and their capacity to modulate intestinal form and function, and described his transcriptomic study to examine the molecular bases underlying the rapid postfeeding metabolic, morphological, and functional responses of the Burmese python intestine.

## Snake venom evolution

**Stephen Mackessy (University of Northern Colorado, USA)** opened up the session on snake venom evolution with a broad discussion of snake venom composition, and the evolution of genes that encode snake venom toxins. The utility of using combined proteomic and genomic approaches to understanding the complex interaction of factors which determine the expression of venom proteins was advocated, because both transcriptional and translational regulatory mechanisms affect final venom composition. This integrated approach can provide important clues for both drug discovery and more effective treatment of snakebite cases. **Darin Rokyta (Florida State University, USA)** discussed plans to utilize a high-throughput comparative transcriptomic approach for studying the evolution of venom-gene repertoires, showing preliminary evidence for the ability of the approach to identify differences between venom-gland transcriptomes of individuals and species. The potential of such data for identifying examples of local adaptation and purifying or positive selection, and for the de novo characterization of alternative-splicing patterns, was examined. **Kenneth Wray (Florida State University, USA)** followed with a detailed analysis of the experimental design of the overall venom-evolution project and the identification of genes under positive selection. The utility for applying transcriptomic data to questions concerning coevolution of predators and prey was also discussed.

## Summary

We expect the extensively annotated complete genome of the Burmese Python will provide a critical scaffold from which the genomes of other snakes (and reptiles) can compared and annotated. Together with the completion of the high-quality King Cobra genome and draft blind snake genome, model species representing the three major clades of snakes will be available as a first core set of reference genomes from which future snake genomics efforts may be effectively launched. Applications of these first three snake genomes will include a better understanding of extreme physiological adaptation in snakes, with applications to the human condition, and will reveal decisive information concerning the evolution of venom toxins from ancestral non-toxin proteins. This small focused-topic conference proved extremely important for driving the field forward, and coordinating efforts and collaborative work across a broad set of international researchers. We plan on future annual conferences to continue these collaborative coordinated efforts, and welcome the inclusion of others. Updated information on progress of the group’s sequencing efforts will be communicated via www.snakegenomics.org.

